# Supplementary material for: Overexpression of CDC20 Confer a Poorer Prognosis in Bladder Cancer Identified by Gene Co-Expression Network Analysis
Source: Diagnostics (Basel). 2025 Nov 27;15(23):3016. doi: 10.3390/diagnostics15233016 (PMC12691489; doi:10.3390/diagnostics15233016)
Supplement: Supplementary file 1 [file diagnostics-15-03016-s001.zip › Supplementary File 2-TableS1Reagents.pdf]

Reagents	Manufacturer	catalog numbers
Roswell Park Memorial Institute (RPMI) 1640 Medium	Scientific Cells, San Diego, CA	SC-RPMI-100
fetal bovine serum	Gibco, Grand Island, NY	26140079
Lipofectamine 2000	Invitrogen, USA	11668019
TRIzol	Invitrogen, USA	15596026
iScript cDNA Synthesis Kit	Bio-Rad, USA	1708891
Cell Counting Kit-8 (CCK-8) Kit	Vazyme, China	K1018
