# Supplementary material for: Overexpression of CDC20 Confer a Poorer Prognosis in Bladder Cancer Identified by Gene Co-Expression Network Analysis
Source: Diagnostics (Basel). 2025 Nov 27;15(23):3016. doi: 10.3390/diagnostics15233016 (PMC12691489; doi:10.3390/diagnostics15233016)
Supplement: Supplementary file 1 [file diagnostics-15-03016-s001.zip › Supplementary File 4-TableS3.pdf]

Table 1: Clinical characteristic of bladder cancer patients with high and low expression of CDC20

Characteristic		Low expression of CDC20	High expression of CDC20	*P*
Total, n		207	207	
Age, n (%)	<=70	124 (30%)	110 (26.6%)	0.197
	>70	83 (20%)	97 (23.4%)	
Gender, n (%)	Female	52 (12.6%)	57 (13.8%)	0.655
	Male	155 (37.4%)	150 (36.2%)	
Histologic grade, n (%)	High Grade	186 (45.3%)	204 (49.6%)	< 0.001
	Low Grade	20 (4.9%)	1 (0.2%)	
Pathologic stage, n (%)	Stage I	3 (0.7%)	1 (0.2%)	0.570
	Stage II	68 (16.5%)	62 (15%)	
	Stage III	66 (16%)	76 (18.4%)	
	Stage IV	69 (16.7%)	67 (16.3%)	
T stage, n (%)	T1	3 (0.8%)	2 (0.5%)	0.117
	T2	59 (15.5%)	60 (15.8%)	
	T3	90 (23.7%)	106 (27.9%)	
	T4	38 (10%)	22 (5.8%)	
N stage, n (%)	N0	117 (31.6%)	122 (33%)	0.153
	N1	18 (4.9%)	28 (7.6%)	
	N2	46 (12.4%)	31 (8.4%)	
	N3	4 (1.1%)	4 (1.1%)	
M stage, n (%)	M0	117 (54.9%)	85 (39.9%)	1.000
	M1	6 (2.8%)	5 (2.3%)	
Lymph vascular invasion, n (%)	No	56 (19.8%)	74 (26.1%)	0.008

	Yes	91 (32.2%)	62 (21.9%)	
Primary therapy outcome, n (%)	PD	29 (8.1%)	41 (11.5%)	0.289
	SD	15 (4.2%)	16 (4.5%)	
	PR	12 (3.4%)	10 (2.8%)	
	CR	127 (35.6%)	107 (30%)	

This table presents the distribution of clinicopathological characteristics between bladder cancer patients with low and high CDC20 expression (n=414). Statistical analysis was performed using appropriate tests (Chi-square or Fisher's exact test) to compare categorical variables between the two groups.

1. **Significant Associations:**
    - **Histologic grade**: A strong association was observed (p<0.001), with high-grade tumors showing substantially higher CDC20 expression (49.6% vs 45.3%) and low-grade tumors being predominantly in the low CDC20 expression group (4.9% vs 0.2%).
    - **Lymph vascular invasion**: A significant association was found (p=0.008), with the low CDC20 expression group showing higher incidence of lymph vascular invasion (32.2% vs 21.9%).
2. **Non-significant Associations:**
    - No significant differences were found in age distribution (p=0.197), gender (p=0.655), pathologic stage (p=0.570), T stage

(p=0.117), N stage (p=0.153), M stage (p=1.000), or primary therapy outcome (p=0.289) between CDC20 expression groups.
