# Supplementary material for: Overexpression of CDC20 Confer a Poorer Prognosis in Bladder Cancer Identified by Gene Co-Expression Network Analysis
Source: Diagnostics (Basel). 2025 Nov 27;15(23):3016. doi: 10.3390/diagnostics15233016 (PMC12691489; doi:10.3390/diagnostics15233016)
Supplement: Supplementary file 1 [file diagnostics-15-03016-s001.zip › Supplementary File 5-TableS4.pdf]

Table 2: Multivariate Cox Regression Analysis of Factors Associated with Overall Survival in Bladder Cancer Patients

Characteristics	Total(n)	Odds Ratio (OR)	P value
T stage (T3&T4 vs. T1&T2)	380	1.000 (0.651-1.536)	1.000
N stage (N1&N2&N3 vs. N0)	370	0.889 (0.579-1.361)	0.587
M stage (M1 vs. M0)	213	1.147 (0.321-3.929)	0.825
Histologic grade (High Grade vs. Low Grade)	411	21.935 (4.505-395.580)	0.003
Pathologic stage (Stage III&Stage IV vs. Stage I&Stage II)	412	1.194 (0.790-1.806)	0.400

This table presents the results of univariate logistic regression analysis examining the association between CDC20 expression levels and key clinicopathological characteristics in bladder cancer patients. The analysis reveals one statistically significant finding among the evaluated parameters.

Key Observations:

- Histologic grade demonstrates a strong positive association with CDC20 expression (OR=21.935, 95% CI: 4.505-395.580, p=0.003), indicating that high-grade tumors have approximately 22 times higher odds of elevated CDC20 expression compared to low-grade tumors.
- No statistically significant associations were observed between CDC20 expression and conventional TNM staging parameters, including T stage (p=1.000), N stage (p=0.587), M stage (p=0.825), or overall pathologic stage (p=0.400).

Methodological Notes:

1. Analysis was performed using univariate logistic regression models.
2. Effect sizes are presented as odds ratios with 95% confidence intervals.
3. Statistical significance was defined as $p<0.05$.
4. Variation in sample sizes across characteristics reflects data availability in the cohort.
